# Editorial: Executive functions and language processing in persons with aphasia

**DOI:** 10.3389/fpsyg.2023.1183870

**Published:** 2023-03-28

**Authors:** Maria Andreou, Eleni Peristeri, Spyridoula Varlokosta

**Affiliations:** ^1^Department of Speech and Language Therapy, University of Peloponnese, Kalamata, Greece; ^2^Department of Theoretical & Applied Linguistics, School of English, Aristotle University of Thessaloniki, Thessaloniki, Greece; ^3^Department of Linguistics, Faculty of Philology, National and Kapodistrian University of Athens, Athens, Greece

**Keywords:** executive functions, language, Primary Progressive Aphasia, post-stroke aphasia, Alzheimer's Disease

The relationship between executive functions (EFs) and language processing in aphasia is an area of vigorous research. Converging evidence indicates that persons with aphasia (PwA) have deficits in various EFs, including inhibition, working memory (WM), and cognitive flexibility, compared to language-unimpaired individuals (e.g., Murray, 2017; Andreou and Peristeri, 2021). While performance of PwA in language processing tests has been shown to draw on domain-general EFs (e.g., Peristeri et al., 2020), there is also evidence that executive deficits in aphasia are independent of deficits to specific language domains (e.g., Schumacher et al., 2019). Given that the relation between language and EFs remains a matter of debate, the performance of PwA and language-unimpaired individuals on language processing and EF tests might provide valuable insights into the interface between language and domain-general cognition, both from a theoretical and a clinical perspective.

Issues of executive functioning and links to language processing are discussed by Horne et al. The study provides fresh evidence that semantic WM plays a significant role during sentence comprehension in aphasia, especially when the comprehension demands are high, as in the case of object relatives, dative sentences, and embedded passives, while phonological WM may play a backup role across all sentence types independently of structural demands. The study provides support for the interaction of language and domain-general cognition in aphasia, and for the conceptualization of WM in terms of domain-specific models that postulate separate buffers for semantic and phonological verbal information.

The language-cognition divide is also pursued by Büttner-Kunert et al., who show that discourse production is impaired in individuals with chronic traumatic brain injury (TBI) compared to age- and education-matched healthy adults, and that this impairment associates with the individuals' verbal executive dysfunctions and perceived social-communicative participation. Individuals with TBI produced stories with fewer critical components and faced greater difficulty than controls in generating bridging inferences when asked to fill in critical causal gaps in short texts. They also scored lower than controls in EFs assessed through a semantic fluency and category-switching task. Their verbal fluency performance was, in turn, associated with their ability to generate bridging inferences and their communicative self-perception. The findings provide new evidence for an interplay between language and cognitive abilities in chronic TBI, that cannot otherwise be revealed with conventional aphasia tests.

The link between WM and language processing is explored by Arslan et al. in a study that investigates sentence repetition deficits in French-speaking individuals with Primary Progressive Aphasia (PPA) and Alzheimer's Disease (AD) through a sentence repetition span task. Based on the assumption that sentence repetition difficulties in PPA might be related to the WM-intensive nature of verbal processing, known to be affected in the logopenic variant of PPA (lvPPA), the authors explore whether greater difficulties in sentence repetition are encountered in individuals with lvPPA compared to individuals with the semantic variant of PPA (svPPA) or with AD. The study provides intriguing evidence that WM-intensive sentence repetition impairments as measured with a span task design is not a clear sensitive marker that can distinguish lvPPA from other neurodegenerative conditions, like svPPA and AD.

Salmons et al. examine the association between language comprehension and memory deficits in PwA in order to evaluate the hypothesis that the two deficits are due to a common underlying mechanism, namely short-term memory (STM). Experimental evidence from Catalan-speaking individuals with different types of aphasia and non-brain damaged controls indicated lower performance of the former compared to the latter group on tasks assessing visual and verbal STM and sentence comprehension. However, the results also revealed that visual STM was better preserved than verbal STM, and that there were no significant correlations between STM and language comprehension. The authors argue that the lack of significant associations between sentence comprehension and STM does not support a common underlying mechanism responsible for deficits in both domains.

Fyndanis et al. examine the role of WM, speed of processing, and education in verb-related morphosyntactic production in neurologically healthy middle-aged and older participants. The authors demonstrate that education and WM play a significant role in verb-related morphosyntactic production. Their results suggest that verb-related morphosyntactic production is predominantly supported by verbal, domain-specific, but not domain-general, memory resources, and that both the storage and processing components of WM are involved in verb production. The study also demonstrates that verbal WM affects specific morphosyntactic aspects of verb production, including grammatical Aspect and Time Reference/Tense. The results from healthy aging individuals are relevant to persons with non-fluent aphasia, who often exhibit co-occurring verb production and verbal WM or/and STM impairments. As such, impaired verb production of PwA could be due at least partially to their reduced verbal WM or/and STM capacity.

The study by Karpathiou and Kambanaros aims at establishing differences on neuropsychological skills and connected speech production between Greek-speaking individuals with PPA and AD. Participants with PPA differed from participants with AD on linguistic measures, including sentence repetition and picture description, since the PPA group failed to repeat long frequent sentences and produced fewer narrative and unique words while describing pictures compared to the AD group. Moreover, individuals with PPA tended to produce more phonological errors, and shorter and less elaborate sentences in story retelling than individuals with AD. Despite these differences, both groups were equally impaired on verbal EFs. The study highlights areas in neuropsychological testing and narrative analysis that are easy-to-use and promising screens for assessing language and cognitive processes that may in turn contribute to the differential diagnostic process and the assessment of language impairment in PPA and AD.

In sum, the various contributions in this issue serve to elucidate the relation between language and cognition and the precise nature of this relation. The studies demonstrate that the interaction between language and domain-general cognitive control functions is not an all-or-nothing phenomenon, but rather relies on selective properties of language processing. The findings are promising in propelling new research in novel directions that will further inform the language-cognition architecture.

## Author contributions

MA, EP, and SV had an equal contribution to the drafting and editing of the manuscript. All authors contributed to the article and approved the submitted version.

## References

[B1] Andreou M. Peristeri E. (2021). Interference resolution in non-fluent variant primary progressive aphasia: evidence from on-line picture naming. Cogn. Behav. Neurol. 34, 11–25. 10.1097/WNN.0000000000000255 33652466

[B2] Murray L. L. (2017). Design fluency subsequent to onset of aphasia: a distinct pattern of executive function difficulties? Aphasiology 31, 793–818. 10.1080/02687038.2016.1261248

[B3] Peristeri E. Tsimpli I. Dardiotis E. Tsapkini K. (2020). Effects of executive attention on sentence processing in aphasia. Aphasiology 34, 943–969. 10.1080/02687038.2019.1622647 32952261 PMC7500515

[B4] Schumacher R. Halai A. D. Lambon Ralph M. A. (2019). Assessing and mapping language, attention and executive multidimensional deficits in stroke aphasia. Brain 142, 3202–3216. 10.1093/brain/awz258 31504247 PMC6794940

